# Antioxidant metabolism variation associated with alkali-salt tolerance in thirty switchgrass (*Panicum virgatum*) lines

**DOI:** 10.1371/journal.pone.0199681

**Published:** 2018-06-25

**Authors:** Guofu Hu, Yiming Liu, Tianqi Duo, Bingyu Zhao, Guowen Cui, Jing Ji, Xiao Kuang, Erik H. Ervin, Xunzhong Zhang

**Affiliations:** 1 College of Animal Science and Technology, Northeast Agricultural University, Harbin, Heilongjiang Province, China; 2 Department of Crop and Soil Environmental Science, Virginia Tech, Blacksburg, VA, United States of America; 3 Tropical Crops Genetic Resources Institute, Chinese Academy of Tropical Agricultural Sciences (CATAS) / Key Laboratory of Crop Gene Resources and Germplasm Enhancement in Southern China, Ministry of Agriculture, Hainan Danzhou, P.R. China; 4 Department of Horticulture, Virginia Tech, Blacksburg, VA, United States of America; 5 Department of Plant and Soil Sciences, University of Delaware, Newark, DE, United States of America; Huazhong Agriculture University, CHINA

## Abstract

Soil salinization is a major factor limiting crop growth and development in many areas. Switchgrass (*Panicum virgatum* L.) is an important warm-season grass species used for biofuel production. The objective of this study was to investigate antioxidant metabolism, proline,and protein variation associated with alkali-salt tolerance among 30 switchgrass lines and identify metabolic markers for evaluating alkali-salt tolerance of switchgrass lines. The grass lines were transplanted into plastic pots containing fine sand. When the plants reached E5 developmental stage, they were subjected to either alkali-salt stress treatment (150 mM Na^+^ and pH of 9.5) or control (no alkali-salt stress) for 20 d. The 30 switchgrass lines differed in alkali-salt tolerance as determined by the level of leaf malondialdehyde (MDA), antioxidant enzyme activity [(superoxide dismutase (SOD), catalase (CAT), ascorbate peroxidase (APX)], proline and protein. Alkali-salt stress increased MDA, proline, SOD, reduced CAT activity, but its effect on protein and APX varied depending on lines. Wide variations in the five parameters existed among the 30 lines. In general, the lines with higher CAT activity and lower proline content under alkali-salt stress had less MDA, exhibiting better alkali-salt tolerance. Among the five parameters, CAT can be considered as valuable metabolic markers for assessment of switchgrass tolerance to alkali-salt stress.

## Introduction

Salt-alkalization is becoming a major environmental and land resource problem [1]. Soil salinization-caused the loss of agricultural land accounts for 0.25 to 0.5 Mha each year in the world [1]. Soil salinization and alkalization significantly reduce crop productivity [2–4]. There are about 950 million ha of saline-alkalized land worldwide [5]. Therefore, revegetation for this kind of lands and corresponding plant screening will be particularly important. Both saline and alkaline were most important stress factors to plant growth and development [6, 7]. Usually saline stress is associated with high level of ion predominantly in the form of NaCl and Na_2_SO_4_ which can interrupt ion homeostasis and balance in plants [6, 7], cause osmotic stress and ion overload [8–10]. In contrast, the effects of alkaline salt stress are mainly associated with high salinity, high pH, and their interactions, because of a high level of NaHCO_3_ and Na_2_CO_3_ found in soil solution at high pH levels [3]. Soil alkali-salinity negatively affects seed germination and plant growth and development [11, 12].

Plants have developed various defense systems to cope with abiotic stress, such as antioxidant defenses, hormonal regulation, osmotic adjustment, and saturation level of cell membrane lipids [13]. Salt stress may induce cellular changes due to accumulation of toxic compounds in cells including reactive oxygen species (ROS) such as superoxide radical (O_2_^-^), hydrogen peroxide (H_2_O_2_), hydroxyl radical (-OH) and singlet oxygen(O_2_) The antioxidant enzyme SOD converts superoxide radicals into H_2_O_2_, which is further scavenged by POD and by CAT [14, 15]. APX uses ascorbate as reductants to reduce H_2_O_2_ to water in various cell compartments [14, 15]. Plants have developed antioxidant defense systems to prevent or reduce damage due to these ROS and protect cells, but activities of a series of antioxidant enzymes and reducing substances varied between species [16].

Many studies indicated that the antioxidant enzyme activity is correlated with plant salt stress tolerance [14, 17, 18]. However, little study was reported on activity of antioxidant enzymes under salt stress with high pH in switchgrass. Therefore, investigating the antioxidant metabolism responses to alkaline salt stress is important for understanding the underlying mechanism of plant tolerance to alkaline salt stress.

Switchgrass (*Panicum virgatum* L.) is a warm-season perennial grass species native to North America [19–21]. Switchgrass requires relatively modest level of fertility, therefore, it can grow well on marginal land, which includes millions of hectares of salt-alkalized lands [8, 22–24]. It can also be used for soil conservation because of its strong root system [25]. Switchgrass is not only used for forage [22, 23], but also for large scale lignocellulosic biofuel production [26, 27].

Switchgrass has a great application potential for revegetation of salt-alkalized lands, so it is important to investigate alkali-salt resistant mechanism for switchgrass. Hu *et al*.[28] evaluated alkali-salt stress tolerance of 30 switchgrass lines and found that a large variation existed among the 30 lines in response to alkali-salt. The results by Hu *et al*.[28] showed that alkali-salt stress (150 mM Na^+^, pH 9.5) damaged cell membrane and reduced the RWC, Pn, gs, and Tr in switchgrass. Hu *et al*.[28] found that selected lowland lines, including Alamo, TEM-SEC, TEM-SLC, and Kanlow, were alkali-salt tolerant. In contrast, three lowland lines, including AM-314/MS-155, BN-13645-64, and two upland lines, including Caddo and Blackwell-1, were alkali-salt sensitive. These results suggest that the switchgrass lines varied widely in alkali-salt stress tolerance, but adjustment mechanism of antioxidant enzymes under alkali-salt stress for switchgrass has not been reported.

The objective of this study was to investigate antioxidant metabolism, proline and protein variation associated with alkali-salt tolerance among 30 switchgrass lines and identify metabolic markers for evaluating alkali-salt tolerance of switchgrass lines. Results from this study could be helpful in enabling accommodation of switchgrass to alkali-salt stress, improving the quality of anti-alkali-salt switchgrass and exploiting saline-alkalized land eventually.

## Materials and methods

### Plant materials and growth conditions

This study was conducted in the greenhouse of Virginia Tech, Blacksburg, VA, USA. Thirty experimental lines used in this study included Alamo, AM-314/MS-155, BN-13645-64, TEM-SEC, Kanlow, TEM-LoDorm, TEM-SLC, 70SG0019, Blackwell-1, Blackwell-3, BN-8624-67, Caddo, Cave-in-Rock, 70SG0023, T16971, 70SG001, BN-12323-69, 70SG002, BN-18758-67, 70SG0018, T-2086, Grenville-2, Sunburst, BN-11357-63, Summer, 70SG0022, BN-10860-61, Grif Nebraska 28, Pathfinder, and Dacotah. The seven lines (Alamo, AM-314/MS-155, BN-13645-64, TEM-SEC, Kanlow, TEM-LoDorm, and TEM-SLC) were lowland ecotypes, while the rest of them were upland ecotypes.

The 30 lines were obtained from the United States Department of Agriculture Germplasm Center and maintained in the greenhouse facility of Virginia Tech, Blacksburg, Virginia. About 12 tiller buds of similar size (about 15 cm tall) from each line, which were grown in the greenhouse, were transplanted into plastic pots (17 cm diameter and 20 cm deep, with eight holes for drainage at the bottom) containing fine sand on Jan. 21, 2014. We placed a piece of plastic screen in the bottom of the pot to prevent sand from leaching out [28]. The grass was grown in the greenhouse with temperature of 26±1°C/ 20±1°C (day/night), photosynthetic active radiation (PAR) at 400 μmol ^-2^ s ^-1^, and a 14 h photoperiod. The seedlings were watered every day and fertilized two times per week with half-strength Hoagland’s solution [29].

The alkali-salt treatment solution, which made of equal molarity Na_2_CO_3_ and NaHCO_3_, with Na^+^ at 150 mM and pH of 9.5, was prepared with 1/2 strength Hoagland’s nutrient solution. The solution for control group was1/2 strength Hoagland’s. In order to avoid stress shock [30], the concentration of alkali-salt solution was applied at 50 mM on the first day (22 March, 2014) and then 100 mM next day, and finally reached 150 mM at third day. Then each pot was placed in a plastic tray (16 cm diameter, 6.5 cm deep) filled with either the alkali-salt solution or 1/2 strength Hoagland’s nutrient solution only. The lower third portions of the pots were submerged in the solutions consistently. Half strength Hoagland’s nutrient solution was added every day to maintain the same level of solution in the tray. Six days after initiation of alkali-salt treatment, leaf samples were taken for analysis of antioxidant enzyme activity, proline, protein, and MDA. We observed that some alkali-salt sensitive lines were injured, and tolerant ones were not severely injured till day 20. Therefore, we took leaf samples at day 6 and completed the experiment at day 20 (10 April, 2014).

### Measurements

We took all measurements at sixth day after alkali-salt stress initiation. Some lines wilted at day 6 and were severely damaged due to alkali-salt stress after day 10, therefore, we did not take measurement after day 6.

#### Leaf malondialdehyde (MDA)

Cell membrane lipid peroxidation was determined based on MDA content. The MDA was measured according to Hodges *et al* [31] with modifications. Leaf samples (0.25 g) were homogenized in10 mL 10% trichloroacetic acid (TCA) and centrifuged at 12,000 g_n_ for 20 min. Then 2 mL 0.6% thiobarbituric acid (TBA) in 10% TCA was added to 2 mL supernatant. The mixture was heated to 100°C in boiling water for 30 min then quickly cooled in an ice bath. After centrifugation at 1600 g_n_ for 10 min, the absorbance of the mixture was measured at 532 and 600 nm. Nonspecific absorbance at 600 nm was subtracted from that at 532 nm. The concentration of MDA was calculated using the MDA’s extinction coefficient of 155 mM^-1^cm^-1^.

#### Leaf proline and soluble protein content

Leaf proline content was estimated according to the colorimetric procedure of Bates et al. [32]. Leaf soluble protein content was extracted and determined according to the method of Bradford [33] using bovine serum albumin (BSA) as the standard with some modification. Frozen leaf samples (0.25 g) were ground in liquid N_2_ and extracted in 3 mL of ice-cold 50 mmol sodium phosphate buffer (pH 7.0) containing 0.2 mM EDTA and 1% polyvinylpyrrolidone (PVP) in an ice-water bath. The homogenate was centrifuged at 12,000 gn for 20 min at 4°C. The supernatant was used to measure protein content. Leaf soluble protein content was extracted and determined according to the method of Bradford [33] using bovine serum albumin (BSA) as the standard with some modification.

#### Leaf antioxidant activity

The supernatant for protein assay was also used for analysis of antioxidant enzyme activity. The superoxide dismutase (SOD) activity was determined by measuring its ability to inhibit the photochemical reduction of nitro blue tetrazolium (NBT) according to the method of Giannopolitis and Ries [34] with minor modifications. The reaction solution (1 mL) contained 50 mM phosphate buffer (pH 7.8), 0.1 mM EDTA, 13 mM methionine,65 μM NBT and 1.3 μM riboflavin, and 30 μL extract. The solution containing no enzyme extract was used as the control. Test tubes were irradiated under fluorescent lights 60 μmol·m^-2^·s^-1^) at 25°C for 10 min. The absorbance of each sample was measured at 560 nm using a spectrophotometer, and one unit of enzyme activity was defined as the amount of enzyme that would inhibit 50% of NBT photoreduction.

Activities of catalase (CAT) was determined using the method of Chance and Maehly [35] with modifications. For CAT, the reaction solution (1 mL) contained 50 mM phosphate buffer (pH 7.0), 15 mM H2O2, and 30 μL of extract. The reaction was initiated by adding the enzyme extract. Changes in absorbance at 240 nm were read in 1 min using a spectrophotometer (ϵ = 39.4 M^-1^cm^-1^).

The activity of ascorbate peroxidase (APX) was detected using the method of Zhang *et al* [36]. The reaction solution (1 mL) contained 50 mM phosphate buffer (pH 7.0), 0.5 mM ascorbate, 0.1 mM EDTA and 100 μL extract. The reaction was started with addition of 10 μL 10 mM H2O2. The absorbance of the solution was determined at 290 nm after 1 min (ϵ = 2.8 mM^-1^cm^-1^).

### Alkali-salt tolerance trait index (ASTTI)

The STTI (salt tolerance trait index) has been used to assess the salt tolerance of different lines in past studies. STTI was calculated using the formula: STTI = (Value of trait under salt stress condition)/ (Value of trait under controlled condition) × 100 [37, 38]. Based on the concept of STTI, ASTTI (Alkaline-salt tolerance trait index) was used to estimate alkali-salt tolerance in this study [28].

### Experimental design and statistical analysis

Eight pots with identical plant growth rate from each line were used for the alkali-salt stress experiment when plants reached the E5 development stage [39]. A split plot design was used with four replications. The main plots consisted of alkali-salt stress treatment and control (no alkali-salt stress). The alkali-salt stress and control groups were physically paired each other and randomly placed in each of four pairs (replicates). The subplots consisted of 30 switchgrass lines. The 30 lines in each main plot were randomly placed in each replicate [28]. All data were analyzed using analysis of variance. Mean separations were calculated using Fisher's protected least significant difference (LSD) test at 5% level. A linear regression equation was established for each trait. Correlations were performed for all traits. All statistical analyses and calculations were completed using SAS 9.0 software (SAS Institute Inc., Cary, NC).

## Results

The main effects alkali-salt stress treatment and line as well as their interactions were statistically significant (*P* ≤ 0.05) for MDA, SOD, APX, CAT, proline, and Protein. Significant differences in the activity of the antioxidant enzymes were observed among the 30 lines.

### Differential MDA responses of 30 lines to alkali-salt stress

**Fig 1:**
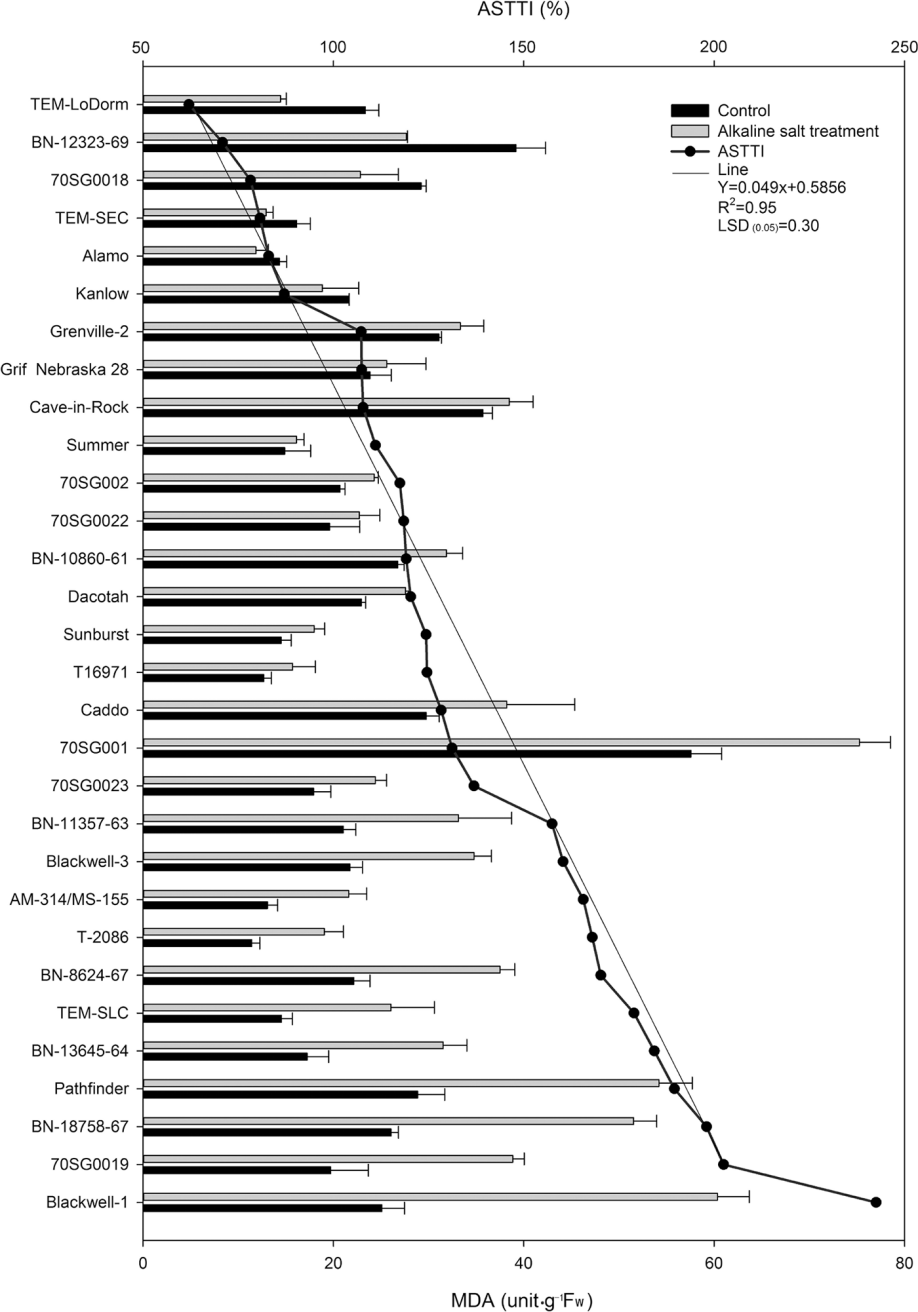
Malondialdehyde (MDA) of 30 switchgrass lines under control (solid bar) and alkali-salt stress (150 mM Na^+^ with pH of 9.5; open bar) at six days of alkali salt treatment. The curve graph represents alkali-salt tolerance trait index (ASTTI). The lower the ASTTI, the better alkali-salt tolerance for switchgrass lines. The line shown an unitary linear regression equation, the greater R^2^ Value indicated better fitting of data. Values are means ±SEM (n = 4). The bar represents LSD (0.05) for ASTTI.

Leaf MDA increased in response to alkali-salt stress treatment in most of the 30 lines. A wide variation was found in MDA response to alkali-salt stress among the 30 lines (Fig 1). Leaf MDA content in the six lines, including TEM-LoDorm, BN-12323-69, 70SG0018, TEM-SEC, Alamo, and Kanlow, remained unchanged or declined in response to alkali-salt treatment. The six lines had ASTTI values less than 87.07%. On the other hand, the greater increase in MDA content was found in Blackwell-1, 70SG0019, BN-18758-67, Pathfinder, BN-13645-64, and TEM-SLC in response to alkali-salt stress. The ASTTI values of these six lines were greater than 178.97%.

### Differential proline responses of 30 lines to alkali-salt stress

**Fig 2:**
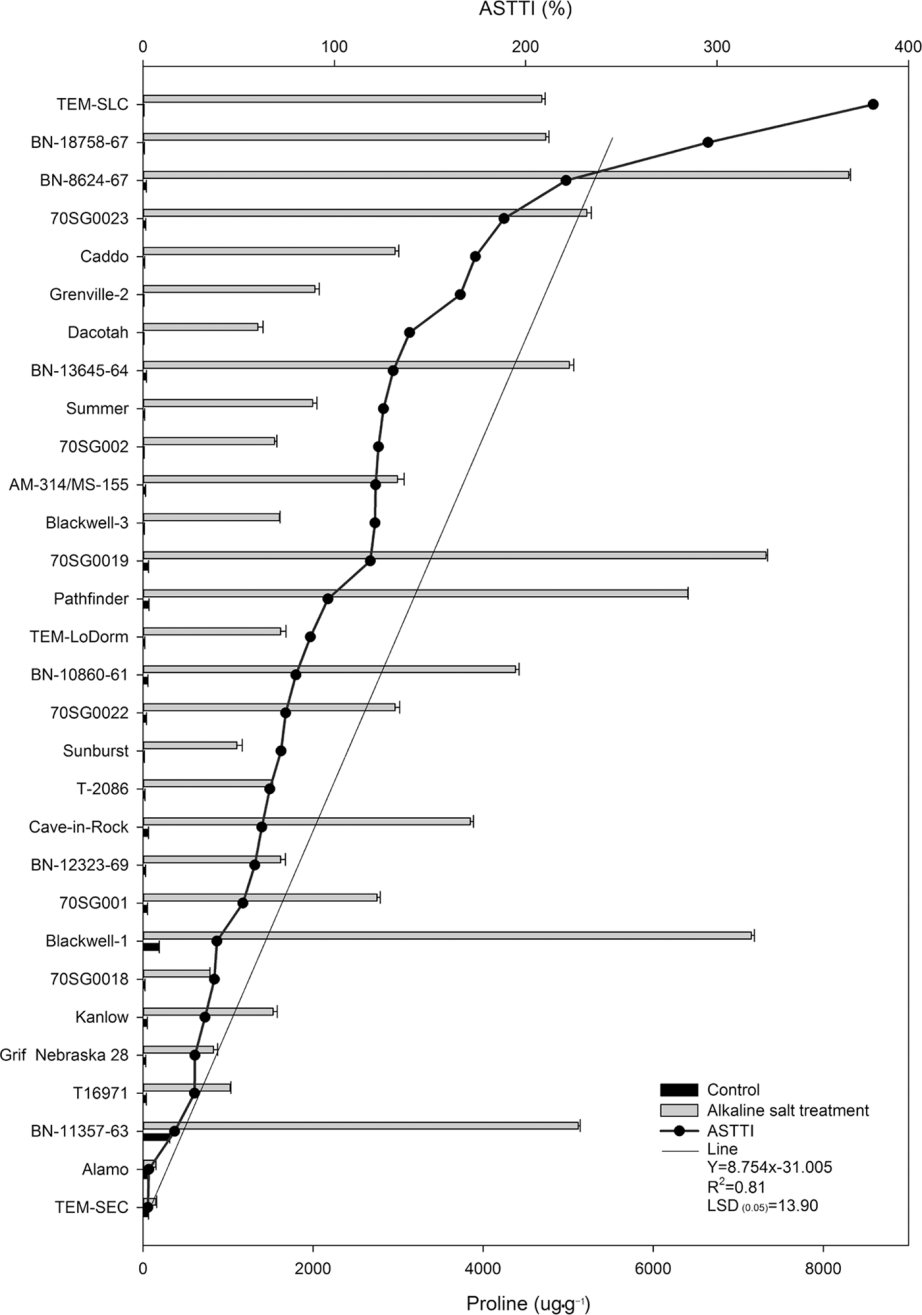
Proline of 30 switchgrass lines under control (solid bar) and alkali-salt stress (150 mM Na^+^ with pH of 9.5; open bar) at six days of alkali salt treatment. **The curve line represents alkali salt tolerance trait index (ASTTI).** The greater the ASTTI, the better the alkali salt tolerance. Values are means ±SE (n = 4). The bar represents LSD (0.05) for ASTTI.

Alkali-salt stress increased leaf proline content regardless of lines (Fig 2). The 30 lines exhibited a wide variation in proline change under alkali-salt stress. The six lines, including TEM-SEC, Alamo, BN-11357-63, T16971, Grif Nebraska 28 and Kanlow, had a relatively smaller change in proline content in response to alkali-salt stress, with ASTTI values less than 323.7%. The proline content had the least increase in TEM-SEC under alkali-salt stress, and the ASTTI value of TEM-SEC was 252.3%. In contrast, TEM-SLC, BN-18758-67, BN-8624-67, 70SG0023, Caddo, and Grenville-2 had greater increase in proline content in response to alkali stress, with ASTTI values greater than 16582.0%. The increase in proline content was the greatest in TEM-SLC among the lines because of alkali-salt stress. The ASTTI value of TEM-SLC was 38167.4%.

### Differential protein content responses of 30 lines to alkali-salt stress

**Fig 3:**
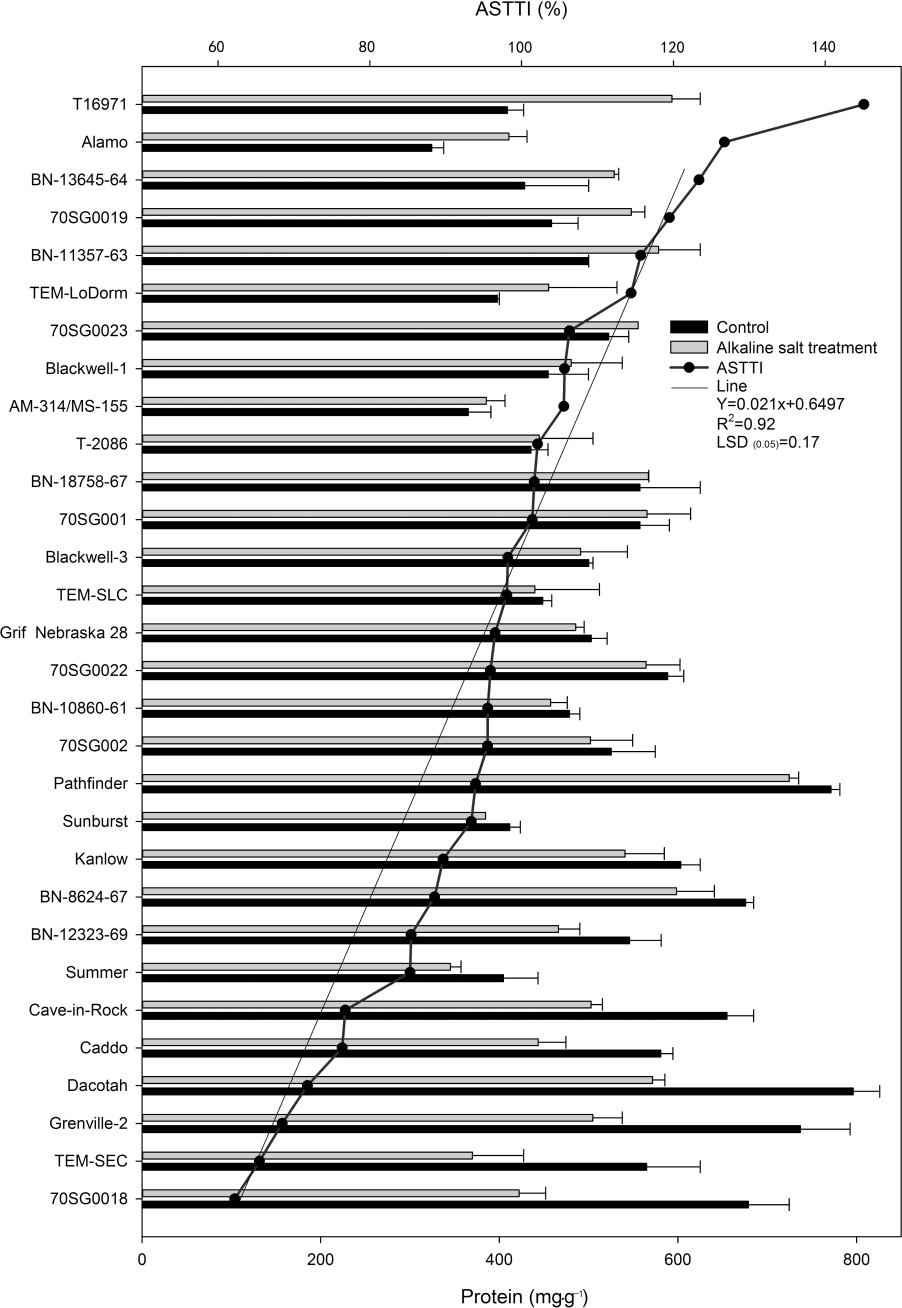
Protein of 30 switchgrass lines under control (solid bar) and alkali-salt stress (150 mM Na^+^ with pH of 9.5; open bar) at 6 days of alkali-salt treatment. The curve line represents alkali-salt tolerance trait index (ASTTI).

Responses of protein to alkali-salt stress varied among on the 30 lines (Fig 3). Alkali-salt stress increased protein content in some lines such as 70SG001, BN-18758-67, T-2086, AM-314/MS-155, Blackwell-1,70SG0023, TEM-LoDorm, BN-11357-63,70SG0019, BN-13645-64, Alamo, and T16971. T16971 had greatest protein increase, with an ASTTI of 145.1%. On the other hand, alkali-salt stress reduced protein in some lines including TEM-SEC, Kanlow, TEM-SLC, Pathfinder, Blackwell-3, BN-8624-67, Cave-in-Rock, BN-12323-69, Caddo, 70SG002, 70SG0018, Sunburst, Summer, Grenville-2, 70SG0022, BN-10860-61, Grif Nebraska 28, and Dacotah.70SG0018 had greatest protein reduction, with an ASTTI of 62.20%.

### Differential SOD responses of 30 lines to alkali-salt stress

**Fig 4:**
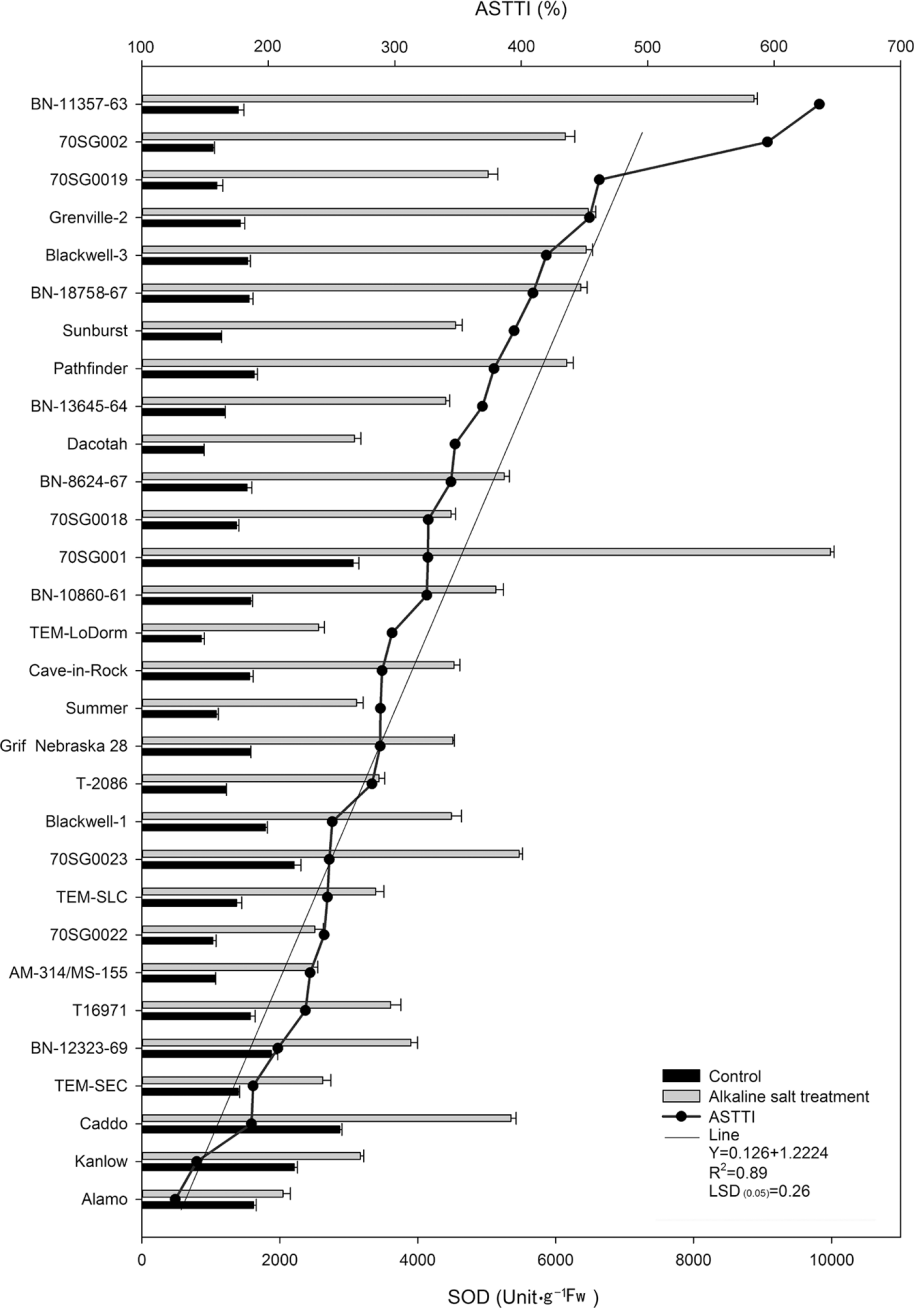
Superoxide dismutase (SOD) of 30 switchgrass lines under control (solid bar) and alkali-salt stress (150 mM Na^+^ with pH of 9.5; open bar) at six days of alkali-salt treatment. The curve line represents alkali-salt tolerance trait index (ASTTI). The greater the ASTTI, the better the alkali salt tolerance. Values are means ±SE (n = 4). The bar represents LSD (0.05) for ASTTI.

Alkali-salt stress increased SOD in all lines (Fig 4). The 30 lines varied in SOD responses under alkali-salt stress. The six lines, including Alamo, Kanlow, Caddo, TEM-SEC, BN-12323-69, and T16971 had less increase in SOD and their ASTTI values were less than 229.3%. The increase in SOD was the least in Alamo among the lines under alkali-salt stress, with an ASTTI value of 126.4%. In contrast, BN-11357-63, 70SG002, 70SG0019, Grenville-2, Blackwell-3, and BN-18758-67 had greater SOD increase under alkali-salt stress, and their ASTTI values were greater than 409.3%. The BN-18758-67 had the greatest increase of SOD with an ASTTI value of 635.8%.

### Differential APX responses of 30 lines to alkali-salt stress

**Fig 5:**
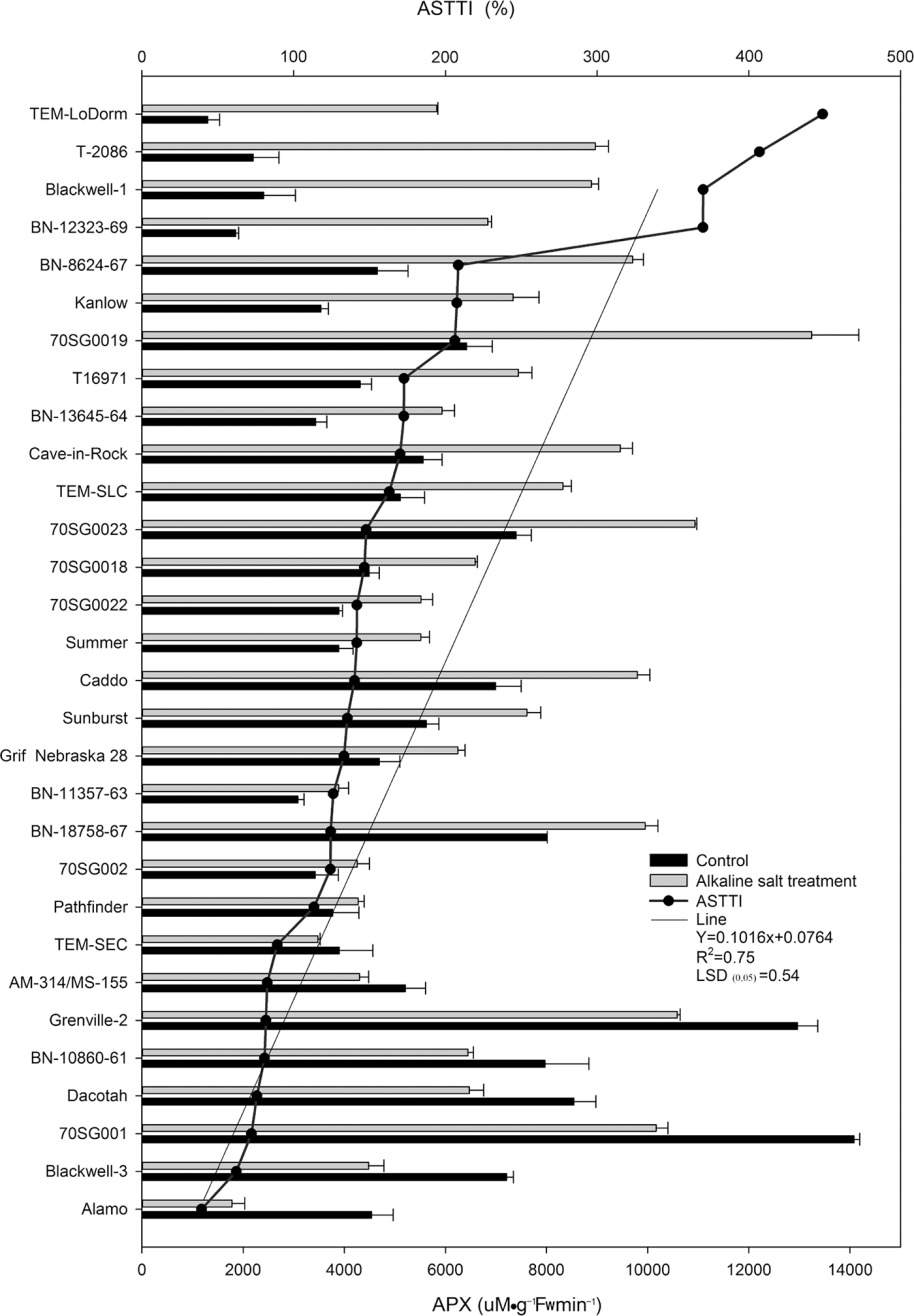
Ascorbate peroxidase (APX) of 30 switchgrass lines under control (solid bar) and alkali-salt stress (150 mM Na^+^ with pH of 9.5; open bar) at six days of alkali salt treatment. The curve line represents alkali-salt tolerance trait index (ASTTI). The greater the ASTTI, the better alkali salt tolerance. Values are means ±SE (n = 4). The bar represents LSD (0.05) for ASTTI.

The APX responses to alkali-salt stress varied greatly among the 30 lines (Fig 5). Alkali-salt stress increased APX of all lines under alkali-salt stress except eight lines including Alamo, Blackwell-3, 70SG001, Dacotah, BN-10860-61, Grenville-2, AM-314/MS-155, and TEM-SEC (Fig 4). Alamo, Blackwell-3, 70SG001, Dacotah, BN-10860-61, Grenville-2, AM-314/MS-155, and TEM-SEC had less APX reduction than the control because of alkali-salt stress, and they had ASTTI values less than 89.2%. The lowest ASTTI value (39.3%) was found in Alamo. Alkali-salt stress increased APX in TEM-LoDorm, T-2086, Blackwell-1, BN-12323-69, BN-8624-67 and Kanlow, with ASTTI values greater than 207.5%. 70SG0019 had the greatest APX, with an ASTTI value of 448.7%.

### Differential CAT responses of 30 lines to alkali-salt stress

**Fig 6:**
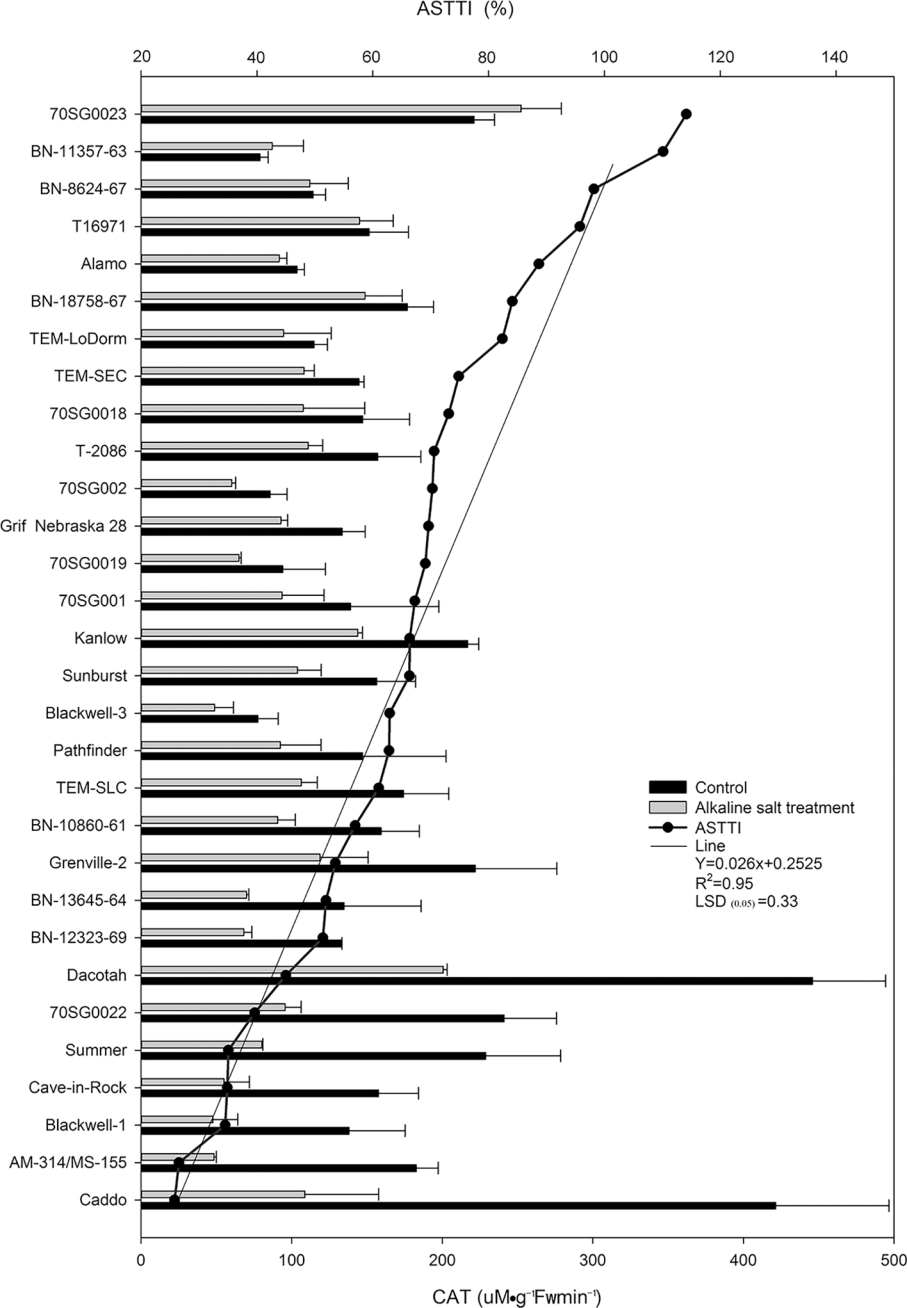
Catalase (CAT) of 30 switchgrass lines under control (solid bar) and alkali-salt stress (150 mM Na^+^ with pH of 9.5; open bar) at 6 days of alkali-salt treatment. The curve line represents alkali-salt tolerance trait index (ASTTI). The greater the ASTTI, the better the alkali salt tolerance. Values are means ±SE (n = 4). The bar represents LSD (0.05) for ASTTI.

A variation in CAT responses to alkali-salt stress was found among the 30 lines. Alkali-salt stress reduced CAT in all lines except 70SG0023 and BN-11357-63 (Fig 6). Caddo, AM-314/MS-155, Blackwell-1, Cave-in-Rock, Summer, and 70SG0022 had greater reduction of CAT because of alkali-salt stress, and they had ASTTI values less than 39.62%. Caddo had the greatest reduction of CAT, with an ASTTI of 25.79%. On the other hand, 70SG0023, BN-11357-63, BN-8624-67, T16971, Alamo, and BN-18758-67 had less CAT reduction under alkali-salt stress, and they had ASTTI values greater than 84.12%. The 70SG0023 and BN-11357-63 had greater CAT than d the control with ASTTI values of 114.16% and 110.14%, respectively. The CAT had greater conformity with R^2^ = 0.9504 by unitary linear regression equation analysis.

### Correlation between metabolic parameters

Correlations between the metabolic parameters from the 30 lines subjected to alkali-salt stress are presented in Table 1. There was a significant correlation of MDA with proline (r = 0.4188, *P*<0.05). Correlation between CAT and protein was also statistically significant (r = 0.3954, *P* <0.05).

**Table 1:** Correlation coefficient (r) among physiological measurements under alkali-salt stress conditions (day 6).

**Parameters**	**MDA**	**Proline**	**SOD**	**APX**	**CAT**	**Protein**
MDA	1.0000					
Proline	0.4188*	1.0000				
SOD	0.3085	0.1384	1.0000			
APX	0.0498	-0.0881	-0.1743	1.0000		
CAT	-0.0583	-0.0284	0.2408	0.0034	1.0000	
Protein	0.3373	-0.0887	0.0456	0.1628	0.3954*	1.0000

* Significant at *P* = 0.05.

### Differences in antioxidant enzyme and metabolites between alkali-salt tolerant lines and sensitive lines

Based the results from our previous study, a comparison was made between the average of four tolerant lines (TEM-SEC, Alamo, TEM-SLC and Kanlow) and four sensitive lines (AM-314/MS-155, BN-13645-64, Caddo and Blackwell-1) [28]. Alkali-salt tolerant lines had higher CAT activity than sensitive lines (Table 2). Alkali-salt tolerant line TEM-SEC had less MDA, proline, and SOD activity, but higher CAT activity than the sensitive line AM-314/MS-155 (Table 3).

**Table 2:** Differences in malondialdehyde (MDA), proline, superoxide dismutase (SOD), ascorbate peroxidase (APX), catalase (CAT), and protein between the average of four tolerant lines and the average of four sensitive lines of switchgrass* (day 6).

Line	MDA	Proline	SOD	APX	CAT	Protein
**Tolerant line**	1.07±0.24a	104.89±68.41a	1.76±0.33a	1.248±0.515a	0.727±0.061a	0.95±0.113a
**Sensitive line**	1.80±0.24a	116.15±68.41a	2.60±0.33a	1.913±0.515a	0.347±0.061b	1.027±0.113a

*Four tolerant lines included TEM-SEC, Alamo, TEM-SLC, and Kanlow; four sensitive lines included AM-314/MS-155, BN-13645-64, Caddo, and Blackwell-1 based on our previous physiological evaluation (Hu et al., 2015).

**Table 3:** Differences in malondialdehyde (MDA), proline, superoxide dismutase (SOD), ascorbate peroxidase (APX), catalase (CAT), and protein between tolerant line of TEM-SEC and sensitive line of AM-314/MS-155 in switchgrass (day 6).

Line	MDA	Proline	SOD	APX	CAT	Protein
**TEM-SEC**	0.81±0.07a	253.9±8.3a	1.88±0.04a	0.908±0.072a	0.748±0.061a	0.939±0.027a
**AM-314/MS-155**	1.66±0.07b	124.1±8.3b	2.33±0.04b	0.831±0.072a	0.267±0.017b	0.956±0.027a
	P = 0.0009	P = 0.0005	P = 0.0016	P = 0.4897	P<0.0001	P = 0.6897

## Discussion

The results of this study indicated that alkali-salt stress (150 mM Na^+^, pH 9.5) caused damage to cell membrane and function as indicated by an increase in MDA a product of lipid peroxidation. Wide variations in MDA responses to alkali-salt stress were found among the 30 switchgrass line. This was consistent with the previous studies by Wang *et al*. [40], Hu *et al*. [28], and Kim *et al*. [41]. In our study, The MDA content increased under alkali-salt stress in all lines except for TEM-LoDorm, BN-12323-69, 70SG0018, TEM-SEC, Alamo and Kanlow (Fig 1). Alkali-salt tolerant line had lower MDA content than sensitive one (Table 3) These lines have been reported to have better alkali-salt tolerant than others based on physiological assessment [28]. This suggests that MDA content and relative change in response to salt stress could be used to measure relative alkali-salt tolerance in switchgrass.

Proline has functions as an osmotic protectant and a free radical scavenger. The results of this study showed that alkali-salt stress induced proline accumulation regardless of lines, with tolerant lines accumulating less proline relative to sensitive lines under alkali-salt stress. This was consistent with the previous study by Kim *et al*.[41]. This suggests that proline level was not positively correlated with salt tolerance in switchgrass [41]. It has been hypothesized that proline catabolism may play an important role in switchgrass tolerance to salt stress and the lines with the highest level of proline may not be able to catabolize proline effectively. It is also possible that nitrogen-rich compound (such as proline) may require more metabolic energy to synthesize than carbon-rich compounds (such as sugars). Therefore, salt tolerant lines may synthesize less proline and more sugars to use metabolic energy more efficiently for osmotic adjustment than sensitive lines, especially under salt stress when photosynthesis and metabolic energy decline. In addition, salt tolerant lines may use more proline to scavenge free radicals or ROS under salt stress, resulting in less proline level when compared to salt-sensitive ones. The results of this study also showed that proline was positively correlated with MDA content (Table 1). The salt tolerant lines, such as TEM-SEC, Alamo, BN-11357-63, T16971, Grif Nebraska 28 and Kanlow had less increase in proline than others, with ASTTI values less than 323.74%. TEM-SEC had the least proline increase under alkali-salt stress, and it had an ASTTI value of 252.31% (Fig 2). This indicates that the salt tolerant line (less MDA) has less proline. Several studies also showed that over accumulation of proline in Arabidopsis could be toxic to cells because of induction of programmed cell death and its change to pyroline-5 carboxylate which is associated with ROS production [42–44]. This suggests that switchgrass lines with less accumulation of proline in response to alkali-salt stress may have better alkali-salt tolerance. Protein responses to alkali-salt stress varied depending on lines. It may not be used for evaluating relative alkali-salt tolerance for switchgrass lines.

Plant antioxidant metabolism is associated with plant tolerance to salt stress [14, 17, 18]. The results of this study showed that alkali-salt stress increased SOD activity regardless of lines (Fig 4). A great variation existed in SOD responses to alkali-salt stress. Alamo, Kanlow, Caddo, TEM-SEC, BN-12323-69 and T16971 had less increase in SOD, with ASTTI values less than 229.34%. Alamo had the least SOD increase among the 30 lines under alkali-salt stress, and its ASTTI was 126.37%. In contrast, BN-11357-63, 70SG002, 70SG0019, Grenville-2, Blackwell-3 and BN-18758-67 had greater SOD increase under alkali-salt stress, with ASTTI values greater than 409.34%. The SOD increased the most for BN-18758-67 with an ASTTI value of 635.83%. The lines with greater increase in SOD activity under salt stress tend to have better alkali-salt tolerance (less MDA). The SOD converts toxic superoxide radical to hydrogen peroxide which is further changed to oxygen and water by CAT or APX. The less increase in SOD in tolerant lines than sensitive ones may indicate that more SOD may be used to remove superoxide radical. The SOD change in response to alkali-salt stress may not be positively correlated with salt stress tolerance in switchgrass.

In the present study, we found that Alkali-salt stress reduced CAT in all lines except 70SG0023 and BN-11357-63 (Fig 6). There were wide variations in CAT responses to alkali-salt stress among the 30 lines. The lines, including Caddo, AM-314/MS-155, Blackwell-1, Cave-in-Rock, Summer and 70SG0022, had greater reduction of CAT because of alkali-salt stress, and their ASTTI values were less than 39.62%. Caddo had the least decline of CAT, with an ASTTI of 25.79%. On the other hand, 70SG0023, BN-11357-63, BN-8624-67, T16971, Alamo and BN-18758-67 had less CAT reduction due to alkali-salt stress, with ASTTI values greater than 84.12%. A significant difference in CAT was found between sensitive line AM-314/MS-155 and tolerance line Alamo (Table 2). In addition, the tolerant line had higher CAT activity than sensitive ones (Table 2). Wang et al. [40] noted that CAT, POD, and SOD are more important than APX, glutathione, and MDA. This suggests that maintaining higher CAT activity under alkali-salt stress may be one of key metabolic markers for identifying lines with greater alkali- salt stress tolerance in switchgrass.

Our results indicated that alkali-salt stress increased APX activity, and a large variation in APX was found among the 30 lines (Fig 4). Alamo, Blackwell-3, 70SG001, Dacotah, BN-10860-61, Grenville-2, AM-314/MS-155 and TEM-SEC had less APX reduction in response alkali-salt stress relative to other lines, with ASTTI values less than 89.20%. Alamo had the least ASTTI of 39.3%. Sensitive lines tended to have higher level of APX than tolerant lines (Table 2). There was no correlation of APX with other five parameters (Table 1). Wang et al. [40] showed that APX, GSH, and MDA are more sensitive to salt stress than SOD, CAT, and POD in switchgrass. This suggests that APX may be used for evaluating alkali-salt tolerance in switchgrass, but less reliable relative to CAT.

In summary, alkali-salt stress increased MDA, proline, SOD, reduced CAT activity, but its effect on protein and APX varied depending on lines. Wide variations in the five parameters existed among the 30 lines. In general, the lines with higher CAT activity and lower proline content under alkali-salt stress had less MDA, exhibiting better alkali-salt tolerance. Among the five parameters, CAT may be considered as valuable metabolic markers for evaluating switchgrass tolerance to alkali-salt stress. The lines or cultivars with higher CAT activity under alkali-salt stress may have greater stress tolerance than those with lower CAT activity.

## Abbreviations

APX: ascorbate peroxidase
AS: alkali salt
ASTTI: alkali-salt tolerance trait index
CAT: catalase
MDA: malondialdehyde
SOD: superoxide dismutase
